# Assessing eligibility for differentiated service delivery, HIV services utilization and virologic outcomes of adult HIV-infected patients in Sierra Leone: a pre-implementation analysis

**DOI:** 10.1080/16549716.2021.1947566

**Published:** 2021-08-18

**Authors:** Sulaiman Lakoh, Darlinda F. Jiba, Alren O. Vandy, Eva Poveda, Olukemi Adekanmbi, Mariama J.S. Murray, Gibrilla F. Deen, Foday Sahr, Christopher J. Hoffmann, Jeffrey M. Jacobson, Robert A. Salata, George A. Yendewa

**Affiliations:** a Department of Medicine, College of Medicine and Allied Health Sciences, University of Sierra Leone, Freetown, Sierra Leone; b Ministry of Health and Sanitation, Freetown, Sierra Leone; c Group of Virology and Pathogenesis, Galicia Sur Health Research Institute (IIS Galicia Sur)-Complexo Hospitalario Universitario De Vigo, SERGAS-UVigo, Spain; d Department of Medicine, College of Medicine, University of Ibadan, Ibadan, Nigeria; e Department of Medicine, University College Hospital, Ibadan, Nigeria; f Department of Medicine, Johns Hopkins School of Medicine, Baltimore, Maryland, USA; g Department of Health, Behavior, and Society, Johns Hopkins Bloomberg School of Public Health, Baltimore, USA; h Department of Medicine, Case Western Reserve University School of Medicine, Cleveland, Ohio, USA; i Division of Infectious Diseases and HIV Medicine, University Hospitals Cleveland Medical Center, Cleveland, Ohio, USA; j Johns Hopkins Bloomberg School of Public Health, Baltimore, Maryland, USA

**Keywords:** HIV, differentiated care, implementation, resource-limited settings, Sierra Leone

## Abstract

**Background:**

There are limited data to help guide implementation of differentiated HIV service delivery (DSD) in resource-limited settings in sub-Saharan Africa.

**Objectives:**

This pre-implementation study sought to assess the proportion of patients eligible for DSD and HIV services utilization, as well as risk factor analysis of virologic failure in Sierra Leone.

**Methods:**

We conducted a retrospective study of adult HIV-infected patients aged 18 years and older receiving care at the largest HIV treatment center in Sierra Leone 2019–2020. Multiple logistic regression was used to identify predictors of virologic failure.

**Results:**

Of 586 unique patients reviewed, 210 (35.8%) qualified as ‘stable’ for antiretroviral therapy (ART) delivery. There was high utilization of certain HIV service programs (e.g. HIV status disclosure to partners (83%) and treatment ‘buddy’ program participation (62.8%)), while other service programs (e.g. partner testing and community HIV support group participation) had low utilization (<50%). Of 429 patients with available viral load, 277 (64.6%) were virologically suppressed. In the multivariate logistic regression analysis of risk factors of virologic failure, CD4 < 350 cells/mm^3^ (p = 0.009), atazanavir-based ART (p = 0.032), once monthly versus once two- or three-monthly ART dispensing (p = 0.028), history of ART switching (p = 0.02), poor adherence (p = 0.001) and not having received adherence support (p < 0.001) were independent predictors of virologic failure.

**Conclusion:**

Approximately one in three HIV-infected patients on ART were eligible for DSD. We identified gaps in HIV care (i.e. low partner testing, treatment ‘buddy’, program participation and a substantially high rate of virologic failure) that need to be addressed in preparation for full implementation of DSD in Sierra Leone.

## BACKGROUND

The global scale-up of antiretroviral therapy (ART) has significantly improved the survival and quality of life of people living with HIV (PLWH) [1]. Despite this, an estimated one-third (12.6 million) of the 38 million PLWH globally did not have access to life-saving treatment in 2019 [1]. To address the treatment gap and maximize the benefit of ART, current guidelines recommend treating all who test positive for HIV, regardless of immunological or clinical status [2–4]. The anticipated increase in treatment demand from adopting the ‘treat all’ strategy is likely to overwhelm already fragile health systems and exacerbate the longstanding financial and human resource constraints thwarting HIV control efforts in low-and-middle-income countries (LMICs). From a service delivery perspective, treatment programs must transform primarily facility-based and physician-led health systems into decentralized and more individualized care models suited to the needs and preferences of the diverse patient populations they must now serve.

The World Health Organization (WHO) and the International AIDS Society (IAS) have provided recommendations to guide treatment programs in LMICs adapt and diversify HIV services using ‘differentiated’ service frameworks [2,5]. Differentiated service delivery (DSD) is a client-centered approach that addresses the health needs of patients taking into account their clinical status, context and preferences [2,5]. Depending on patient characteristics, DSD models vary service intensity (e.g. laboratory monitoring); location (e.g. facility- versus community-based); frequency (e.g. multi-month ART refills) and provider (e.g. task-shifting from physicians or nurses to community health workers) to improve quality of care and health system efficiency [2,5]. Thus, a patient classified as stable may require fewer clinic visits and laboratory monitoring, allowing health care resources to be directed to sicker patients requiring more intensive interventions.

Sierra Leone is one of several LMICs in the West and Central Africa region currently undertaking activities to improve HIV care, in response to specific warnings from the WHO and IAS that this region was lagging behind others in meeting the 90–90-90 goals [6,7]. The national HIV seroprevalence rate of Sierra Leone has remained at 1.5% to 1.7%, yet fewer than 30% of PLWH were receiving ART in 2018, with 26% of those on treatment achieving viral suppression [8]. Studies by our group have identified other entrenched challenges in the local HIV care continuum, suggesting a dynamic epidemic that is characterized by a high prevalence of late-stage presentation and AIDS-related mortality, and rising rates of HIV drug resistance [9–12].

In 2018, the National AIDS Control Program of the Ministry of Health and Sanitation of Sierra Leone released its ‘Guide of Differentiated Care Models in Sierra Leone’, which proposed various DSD models of pediatric and adult HIV care, with ART delivery to clinically stable clients identified as a priority area of focus [13]. Implementation of DSD in Sierra Leone was tentatively set for 2021. To ensure a successful transition, various pilot-level activities that can be considered DSD models have been underway. These include scaling-up of viral load (VL) monitoring and strengthening of client-centered services such as HIV status disclosure and partner testing, targeted adherence counseling and treatment ‘buddy’ and community ART program participation to improve treatment adherence. The advent of the COVID-19 pandemic has necessitated the fast-tracking of DSD rollout plans, which has introduced new implementation complexities warranting the modification of DSD models to support social distancing measures such as fewer clinic visits and multi-monthly ART dispensing [14–18]. However, the overall impact of these pre-implementation measures on HIV service utilization and treatment outcomes have not been fully assessed. Additionally, the proportion of PLWH who qualify for enrollment into specific DSD models remains unknown, making program planning and budgeting difficult for public health experts and policymakers.

The aims of this DSD pre-implementation analysis were to (i) estimate the proportion of adult PLWH receiving care at the largest HIV treatment center in Sierra Leone who meet eligibility criteria for the proposed DSD model for ART delivery to stable clients; (ii) assess the utilization of client-centered services in an attempt to identify potential barriers to service delivery; and (iii) perform a risk factor analysis for virologic failure, an important treatment outcome measure and major criterion for DSD eligibility.

## METHODS

### Study design, site and population

We conducted a retrospective study of adult HIV-infected patients aged ≥18 years who received care at the HIV Clinic at Connaught Hospital in Freetown, Sierra Leone before the onset of the COVID-19 pandemic (October 2019 through February 2020). Connaught Hospital is a 300-bed academic facility that is affiliated with the College of Medicine and Allied Health Sciences of the University of Sierra Leone and is the country’s main referral hospital for adults. The HIV Clinic at Connaught Hospital in Freetown is the largest HIV treatment center in Sierra Leone and provides both outpatient and inpatient HIV services including partner testing and counselling, prevention of mother-to-child transmission and treatment of opportunistic infections.

### Clinical data collection and definitions

Demographic and clinical data were extracted from medical records of HIV-infected patients, who were then classified as clinically stable or unstable using the criteria proposed by the National AIDS Control Program of Sierra Leone (Table 1) [13]. A clinically stable patient was defined as fulfilling all of the following criteria: age 20 years or older, on current ART ≥ 1 year, having no active opportunistic infections` including tuberculosis (TB) in the previous 6 months, adherent to treatment for the previous 6 months, most recent VL < 1000 copies/mL and body mass index (BMI) ≥18.5 kg/m^2^, in addition to health care team not having any concerns about providing longer follow-up intervals for the patient [13]. A patient who failed to meet any one of the aforementioned criteria was classified as an unstable patient (Table 1).

**Table 1.** Classification of adult HIV-infected patients based on DSD criteria for ART delivery in Sierra Leone*

Stable Patient	Unstable patient
*A patient is considered stable if fulfills ALL of the following*: On current ART regimen ≥12 months No active OIs (including TB) in the previous 6 months Adherent for the previous 6 months Most recent viral load <1,000 copies/mL Body mass index ≥18.5 kg/m^2^ Age ≥20 years Healthcare team does not have concerns about providing longer follow-up intervals for the patient	*A patient is considered unstable if has ANY of the following*: On current ART regimen <12 months Any active OIs (including TB) in the previous 6 months Poor or questionable adherence in the previous 6 months Most recent viral load ≥1000 copies/mL Body mass index <18.5 kg/m^2^ Age <20 years Pregnant or breastfeeding Healthcare team has concerns about providing longer follow-up intervals for the patient

*Reproduced from the National AIDS Control Programme, Ministry of Health and Sanitation of Sierra Leone [13].

Antiretroviral therapy, ART; opportunistic infection, OI; tuberculosis, TB.

The WHO has defined virologic failure as VL > 1000 copies/mL based on two consecutive measurements taken at least 3 months apart, with documented adherence support, after taking ART for at least 6 months [2]. However, due to the limited laboratory facilities in most resource-limited settings, various definitions of ‘stable’ clients and virologic failure have emerged, with several DSD models in SSA including the proposed models for Sierra Leone defining virologic failure as a single measurement of VL > 1000 copies/mL obtained at least 12 months after ART initiation [5,13].

Antiretroviral drug substitution was defined as replacing one antiretroviral drug with another, while ART switching was defined as replacing the entire regimen of all three drugs. The commonest reasons recorded for ART substitution were suspected/confirmed drug toxicities and for preventing drug–drug interactions (e.g. substituting boosted-protease inhibitors with efavirenz, EFV, in patients receiving treatment for TB). On the other hand, ART switching was commonly carried out in the event of suspected virologic failure – HIV genotyping is currently not available in Sierra Leone to confirm the presence of drug resistance mutations.

Self-reporting of missed doses, pill counts and reviewing pharmacy refill records and clinic attendance registers were used to access treatment adherence. The Adherence Index was then calculated using the formula [(Total number of pills taken)÷(Total number of pills prescribed)] x100%. Good adherence was defined as an adherence index > 95%.

Alcohol use was defined as consuming > 20 g or 2 drinks of an alcoholic beverage daily, while illicit drug use was defined as using any quantity of any of marijuana, cocaine or injected heroin in the last 30 days.

### Statistical analyses

Statistical analyses were performed using the SPSS Version 25.0 (Armonk, NY; IBM Corp). Categorical variables were reported as frequencies (percentages) and associations assessed using Pearson’s chi-square or Fisher’s exact tests. Continuous variables were presented as medians (interquartile ranges, IQR) and associations assessed using the non-parametric independent samples Mann–Whitney U-test. A logistic regression model was used to identify predictors of virologic failure. Risk factors known to be associated with virologic failure were accessed in the univariate analysis. These included age, gender, educational level, employment status, relationship status, immunologic status (CD4 count), ART regimen based on drug class, history of ART substitution or switching, treatment duration, level of treatment adherence, targeted adherence support and counselling received, comorbidities (history of tuberculosis) and social risk factors (alcohol and illicit drug use). Other than age and gender which were included *a priori*, only variables that attained a p-value of <0.2 in the univariate analysis were included in the multivariate regression model. Associations were reported as crude (OR) and adjusted odds ratios (aOR) with 95% confidence intervals (CI). Differences were considered statistically significant when p was <0.05.

## RESULTS

### Proportion of HIV-infected patients eligible as clinically stable for ART delivery

**Figure 1.**
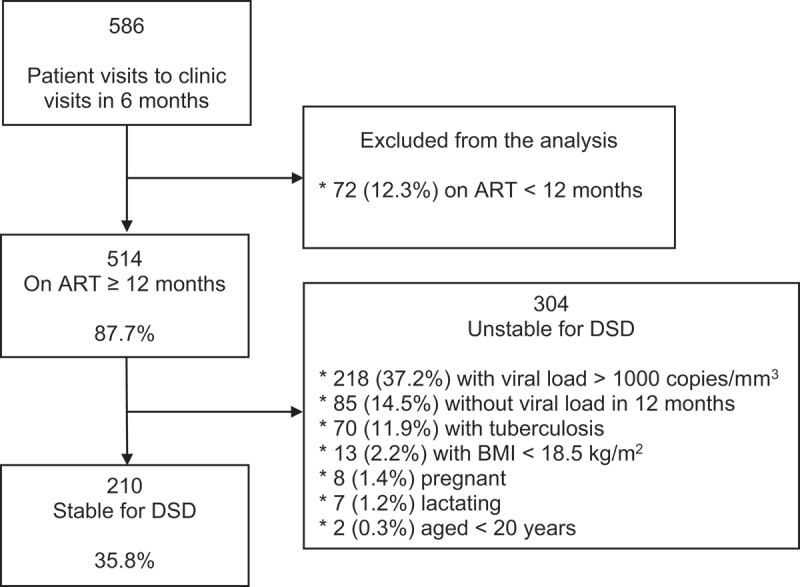
Estimation of proportion of stable HIV-infected clients qualifying for DSD model for ART delivery at Connaught Hospital in Freetown, Sierra Leone

Medical records of 586 unique patient visits to the HIV Clinic at Connaught Hospital during October 2019 through February 2020 were available for review, which was representative of the 6-monthly clinic attendance rate (Figure 1). Of these, 12.3% (72/586) were diagnosed and initiated on ART for less than 12 months and were therefore excluded from the analysis. The DSD eligibility criteria (Table 1) were then applied to the remaining 87.7% (514/586) who were on ART for at least 12 months, of which 35.8% (210/586) met the eligibility criteria as stable for ART delivery, while 51.9% (304/586) were ineligible. The commonest reasons for DSD ineligibility (not mutually exclusive) were VL > 1000 copies/mL (37.2%, 218/586), being on ART for at least 12 months but not having a VL measurement in the last 12 months (14.5%, 85/586), being on ART for less than 12 months and recent history of TB (11.9%, 70/586).

### Demographic and clinical characteristics of stable and unstable patients

Table 2 displays the demographic and clinical characteristics and HIV service utilization of the 514 patients who were on ART for at least 12 months. The majority of patients were female (75.3%, 387/514), had attained secondary education or higher (56.4%, 290/514) and worked in the informal sector (75.5%, 388/514). About 40% (206/514) were married (40.1%, 206/514). The commonest ART regimens were tenofovir (TDF)- (66.1%, 340/514) and EFV- (57.0%, 293/514) based. The median duration on ART was 6.0 years (IQR 3.0–9.0).

**Table 2.** Demographic and clinical characteristics of clinically stable and unstable patients

**Characteristics**	**Total** (N = 514)	**Stable patients** (N = 210)	**Unstable patients** (N = 304)	**p-Value**
**Gender**				
Male	127 (24.7)	49 (23.3)	78 (25.7)	0.548
Female	387 (75.3)	161 (76.7)	226 (74.3)	
**Age**, *years*				
Median, IQR	39 (32–48)	41 (34–50)	38 (31–47)	**0.010**
< 30	86 (16.7)	24 (11.4)	62 (20.4)	
30–39	165 (32.1)	66 (31.4)	109 (35.9)	
40–49	181 (35.2)	64 (30.5)	74 (24.3)	
≥ 50	63 (12.3)	56 (26.7)	59 (19.4)	
**Body mass index**, *kg/m^2^* *(N = 487)*				
Median, IQR	23 (20–26)	24 (21–27)	22 (20–25)	**<0.001**
<18.5 (underweight)	43 (8.8)	-	43 (15.1)	
18.5–24.9 (normal)	271 (55.6)	120 (59.4)	151 (53.0)	
25–29.9 (overweight)	123 (25.3)	60 (29.7)	63 (22.1)	
≥30 (obese)	50 (10.3)	22 (10.9)	28 (9.8)	
**Educational level attained**				
None	135 (26.3)	60 (28.6)	75 (24.7)	0.804
Primary	89 (17.3)	35 (16.7)	54 (17.8)	
Secondary	216 (42.0)	86 (41.0)	130 (42.8)	
Tertiary	74 (14.4)	29 (13.8)	45 (14.8)	
**Occupation**				
Formal sector	42 (8.2)	20 (9.5)	22 (7.2)	0.324
Informal sector	388 (75.5)	161 (76.7)	227 (74.7)	
Unemployed	84 (16.3)	29 (13.8)	55 (18.1)	
**Relationship status**				
Single	154 (30.0)	52 (24.8)	102 (33.6)	0.171
Married	206 (40.1)	88 (41.9)	118 (38.8)	
Divorced	33 (6.4)	14 (6.7)	19 (6.3)	
Widowed	121 (23.5)	56 (26.7)	65 (21.4)	
**Smoking**				
Yes	25 (4.9)	5 (2.4)	20 (6.6)	**0.030**
No	489 (95.1)	205 (97.6)	284 (93.4)	
**Alcohol drinking**				
Yes	52 (10.1)	22 (10.5)	30 (9.9)	0.822
No	462 (89.9)	188 (89.5)	274 (90.1)	
**Illicit drug use**				
Yes	14 (2.7)	3 (1.4)	11 (3.6)	0.134
No	500 (97.3)	207 (98.6)	293 (96.3)	
**History of tuberculosis**				
Yes	70 (13.6)	-	70 (23.0)	**<0.001**
No	444 (86.4)	210 (100)	234 (77.0)	
**Last CD4**, *cells/mm^3^* *(N = 319)*				
Median, IQR	322 (198–509)	355 (217–544)	310 (179–492)	0.089
0–349	175 (54.9)	68 (49.3)	107 (59.1)	
≥350	144 (45.1)	70 (50.7)	74 (40.9)	
**ART regimen by NRTI**				
ABC-based	10 (1.9)	2 (1.0)	8 (2.6)	0.167
AZT-based	164 (31.9)	75 (35.7)	89 (29.3)	
TDF-based	340 (66.1)	133 (63.3)	207 (68.1)	
**ART regimen by PI or NNRTI**				
ATV/r-based	80 (15.6)	9 (4.3)	71 (23.4)	**<0.001**
LPV/r-based	30 (5.8)	10 (4.8)	20 (6.6)	
NVP-based	111 (21.6)	58 (27.6)	53 (17.4)	
EFV-based	293 (57.0)	133 (63.3)	160 (52.6)	
**ART duration**, *years*				
Median, IQR	6.0 (3.0–9.0)	6.0 (3.0–10.0)	6.0 (3.0–6.0)	
< 2	105 (20.4)	32 (15.2)	73 (24.0)	**0.038**
2–5	165 (32.1)	74 (35.2)	91 (29.9)	
6–10	181 (35.2)	72 (34.3)	109 (35.9)	
> 10	63 (12.3)	32 (15.2)	31 (10.2)	
**ART refill frequency**				
1-monthly	241 (46.9)	75 (35.7)	166 (54.6)	**<0.001**
2-monthly	146 (28.4)	61 (29.0)	85 (28.0)	
3-monthly	127 (24.7)	74 (35.2)	53 (17.4)	
**Good ART adherence**				
Yes	445 (86.6)	210 (100)	235 (77.3)	**<0.001**
No	69 (13.4)	-	69 (22.7)	
**Received targeted adherence counselling in last 6 months**				
None	275 (53.5)	141 (67.1)	134 (44.1)	**<0.001**
Once	147 (28.6)	65 (31.0)	82 (27.0)	
Twice	66 (12.8)	4 (1.9)	62 (20.4)	
Three or more times	26 (5.1)	-	26 (8.6)	
**Community HIV support group participation**				
Yes	91 (17.7)	40 (19.0)	51 (16.8)	0.507
No	423 (82.3)	170 (81.0)	253 (83.2)	
**Has treatment ‘buddy’**				
Yes	323 (62.8)	130 (61.9)	193 (63.5)	0.715
No	191 (37.2)	80 (38.1)	111 (36.5)	
**Disclosed HIV status**				
Yes	423 (82.3)	176 (83.8)	247 (81.3)	0.455
No	91 (17.7)	34 (16.2)	57 (18.8)	
**Reasons for nondisclosure** **of HIV status** *(N = 91)*				
Fear of stigmatization	70 (76.9)	28 (80.0)	42 (75.0)	0.784
Fear of discrimination	6 (6.6)	2 (5.7)	4 (7.1)	
Fear of abandonment	6 (6.6)	1 (2.9)	5 (8.9)	
No reason	9 (9.9)	4 (11.4)	5 (8.9)	
**Partner tested for HIV**				
Yes	232 (45.1)	109 (51.9)	123 (40.5)	**0.010**
No	282 (54.9)	101 (48.1)	181 (59.5)	
**Partner HIV status** *(N = 232)*				
Positive	108 (46.6)	53 (48.6)	55 (44.7)	0.551
Negative	124 (53.4)	56 (51.4)	68 (55.3)	
**Most recent viral load**, *copies/mm^3^* *(N = 429)*				
<1000	277 (64.6)	210 (100)	67 (30.6)	**<0.001**
1000–9999	89 (20.7)	-	89 (40.6)	
10,000–99,999	42 (9.8)	-	42 (19.2)	
100,000+	21 (4.9)	-	21 (9.6)	

Antiretroviral therapy, ART; protease inhibitor, PI; nucleot(s)ide reverse transcription inhibitor, NRTI; non-nucleot(s)ide reverse transcription inhibitor, NNRTI.

CD4 count was available for 62.1% (319/514) of patients screened, with a median of 322 cells/mm^3^ (IQR 198–509). VL was available for 82.8% (429/514) of patients, of whom 64.6% (277/429) were virologically suppressed (i.e. VL <1000 copies/mL).

Compared with unstable patients, stable patients were older (median age, 41 versus 38 years; p = 0.01), had higher BMIs (median, 24 versus 22 kg/mm3; p < 0.001) and had higher CD4 counts (median, 355 versus 310 cells/mm^3^; p < 0.001).

### Utilization of HIV services

Overall, 46.9% (241/514) of patients received ART refills monthly, while the remainder had multi-monthly refills. Compared with stable patients, the majority of unstable patients received one-monthly refills (68.9% versus 31.1%; p < 0.001), while most patients who qualified as stable received 3-monthly refills (58.3% versus 41.7%; p < 0.001).

HIV status disclosure was high (82.3%, 423/514), with partner testing and HIV positivity rates of 45.1% (232/514) and 46.6% (108/232), respectively. The commonest reason for HIV status non-disclosure was fear of stigmatization (76.9%, 70/91).

Community HIV support group participation was low (17.7%, 91/514), while nearly two-thirds of patients (63.8%, 323/514) had treatment buddies. ART adherence was estimated as high during the preceding 6 months (86.6%, 445/514). Nearly half of all patients received targeted adherence counselling at least once in the preceding 6 months (46.5%, 239/514).

### Risk factor analysis of virologic failure

Table 3 displays the associations between virologic failure and potential risk factors. After adjusting for confounders in the multivariate regression analysis, there was strong evidence of an association between virologic failure and CD4 < 350 cells/mm^3^ (aOR 2.83, 95% CI [1.29–6.21]; p = 0.009), ritonavir-boosted atazanavir (ATV/r)-based ART compared with EFV-based regimens (aOR 5.46, 95% CI [1.16–25.64]; p = 0.032), one-monthly versus multi-monthly ART dispensing (aOR 2.53, 95% CI [1.11–5.78]; p = 0.028), history of switching ART (aOR 3.97, 95% CI [1.25–12.66]; p = 0.02), history of poor treatment adherence (aOR 10.87, 95% CI [2.75–43.05]; p = 0.001) and not having received targeted adherence counselling during the preceding 6 months (aOR 10.20, 95% CI [4.07–25.64]; p < 0.001).

**Table 3.** Univariate and multivariate analysis of predictors of virologic failure

**Risk factors**	**Virologic outcome**		**Univariate analysis**		**Multivariate analysis**	
	**Failure** (N = 152)	**Suppressed** (N = 277)	**Unadjusted Odds** **Ratio (95% CI)**	**P-value**	**Adjusted Odds** **Ratio (95% CI)**	**P-value**
**Gender**						
Male	35 (23.0)	59 (21.3)	Ref	0.679	Ref	0.512
Female	117 (77.0)	218 (78.7)	0.90 (0.56–1.45)		0.73 (0.28–1.88)	
**Age**, *years*						
< 30	24 (15.8)	40 (14.4)	1.11 (0.64–1.93)	0.708	2.59 (0.95–7.04)	0.062
≥ 30	128 (84.2)	237 (85.6)	Ref		Ref	
**Body mass index**, *kg/m^2^* *(N = 407)*						
<18.5	13 (9.2)	29 (10.9)	0.83 (0.42–1.65)	0.595		
≥ 18.5	128 (90.8)	237 (89.1)	Ref			
**Education**						
None or primary	69 (45.4)	122 (44.0)	1.06 (0.71–1.57)	0.710		
Secondary or tertiary	83 (54.6)	155 (56.0)	Ref			
**Occupation**						
Employed	124 (34.9)	231 (65.1)	Ref	0.634		
Unemployed	28 (37.8)	46 (62.2)	1.13 (0.68–1.90)			
**Relationship status**						
Single	44 (28.9)	75 (27.1)	Ref			
Married	62 (40.8)	115 (41.5)	0.92 (0.57–1.49)	0.732		
Divorced	6 (3.9)	19 (6.9)	0.54 (0.20–1.45)	0.220		
Widowed	40 (26.3)	68 (24.5)	1.00 (0.58–1.72)	0.997		
**Smoking**						
Yes	8 (5.3)	8 (2.9)	1.87 (0.69–5.08)	0.286		
No	144 (94.7)	269 (97.1)	Ref			
**Alcohol/illicit drug use**						
Yes	14 (9.2)	27 (9.7)	0.94 (0.48–1.85)	0.856		
No	138 (90.8)	250 (90.3)	Ref			
**History of tuberculosis**						
Yes	22 (14.5)	40 (14.4)	1.00 (0.57–1.76)	1.000		
No	130 (85.5)	237 (85.6)	Ref			
**Last CD4**, *cells/mm^3^* *(N = 261)*						
<350	54 (68.4)	90 (49.5)	2.21 (1.27–3.85)	**0.005**	2.83 (1.29–6.21)	**0.009**
≥350	25 (31.6)	92 (50.5)	Ref		Ref	
**ART regimen by NRTI**						
ABC-based	7 (4.6)	2 (0.7)	6.76 (1.38–33.33)	**0.019**	1.12 (0.05–23.81)	0.942
AZT-based	54 (35.5)	99 (35.7)	1.05 (0.69–1.60)	0.802	1.46 (0.42–5.13)	0.552
TDF-based	91 (59.9)	176 (63.5)	Ref		Ref	
**ART regimen by PI or NNRTI**						
ATV/r-based	58 (38.2)	10 (3.6)	23.10 (11.00–47.62)	**<0.001**	5.46 (1.16–25.64)	**0.032**
LPV/r-based	15 (9.9)	11 (4.0)	5.43 (2.34–12.66)	**<0.001**	3.70 (0.49–27.78)	0.206
NVP-based	32 (21.1)	69 (24.9)	1.85 (1.09–3.13)	**0.023**	1.46 (0.37–5.78)	0.586
EFV-based	47 (30.9)	187 (67.5)	Ref		Ref	
**ART refill frequency**						
1-monthly	102 (67.1)	92 (33.2)	4.10 (2.69–6.25)	**<0.001**	2.53 (1.11–5.78)	**0.028**
2- or 3-monthly	50 (32.9)	185 (66.8)	Ref		Ref	
**History of ART change**						
Substitution (1 drug)	16 (10.5)	17 (6.1)	3.26 (1.56–6.80)	**0.002**	1.55 (0.29–8.23)	0.610
Switch (2 or more drugs)	69 (45.4)	28 (10.1)	8.56 (5.10–14.29)	**<0.001**	3.97 (1.25–12.66)	**0.020**
No change	67 (44.1)	232 (83.8)	Ref		Ref	
**ART treatment duration**						
< 2	21 (	46 (68.7)	0.96 (0.45–2.04)	0.918		
2–5	46 (	96 (67.6)	1.01 (0.53–1.93)	0.979		
6–10	66 (41.0)	95 (59.0)	1.46 (0.78–2.75)	0.237		
> 10	19 (32.2)	40 (67.8)	Ref			
**Good ART adherence**						
Yes	111 (73.0)	269 (97.1)	Ref	**<0.001**	Ref	**<0.001**
No	41 (27.0)	8 (2.9)	12.35 (5.65–27.03)		10.87 (2.75–43.05)	
**Received targeted adherence counselling in previous 6 months**						
Yes	136 (89.5)	103 (37.2)	Ref		Ref	**<0.001**
No	16 (10.5)	174 (62.8)	14.36 (8.10–25.45)	**<0.001**	10.20 (4.07–25.64)	
**Community HIV support group participation**						
Yes	33	53 (61.6)	1.17 (0.72–1.91)	0.524		
No	119 (34.7)	224 (65.3)	Ref			
**Has treatment ‘buddy’**						
Yes	97 (63.8)	179 (64.6)	0.97 (0.64–1.46)	0.868		
No	55 (36.2)	98 (35.4)	Ref			

Antiretroviral therapy, ART; confidence interval, CI; nucleot(s)ide reverse transcription inhibitor, NRTI; non-nucleot(s)ide reverse transcription inhibitor, NNRTI; protease inhibitor, PI.

## Discussion

This pre-implementation analysis sought to address the paucity of knowledge on HIV service delivery in Sierra Leone as the country prepares to rollout DSD models for ART. We estimated that over one-third (35.8%) of recent adult clinic attendees at the largest HIV treatment center in Sierra Leone met the eligibility criteria as stable clients for DSD. Because differentiating HIV care is a relatively new approach to service delivery in SSA, data on eligibility for DSD models are limited and difficult to compare, given that stability criteria can vary widely across different contexts and settings [2,5]. To further illustrate this point, a recent sub-study from a large randomized trial from two high-prevalence countries (Malawi and Zambia) approximated that 75% (n = 2938) of adult patients screened at 30 ART clinics qualified as stable for ART delivery; however, the eligibility criteria did not include viral suppression, as VL monitoring was not widely available at many of the study sites [19]. Despite these limitations, eligibility estimates could serve as a useful metric for assessing treatment program performance and can help guide program planning, budgeting and funding allocation.

Scaling-up HIV services and optimizing clients’ access are critical to ensuring implementation success; thus, barriers that hinder these processes should be identified and addressed accordingly. Several studies from SSA have emphasized the centrality of reliable and affordable laboratory services for successful navigation of the HIV care continuum [20–22]. In our analysis, we observed gaps in laboratory monitoring, with approximately 62% of patients screened having a CD4 count within the last 12 months while 83% had viral load monitoring within the same time period. Of note, viral load monitoring was first piloted in Sierra Leone in 2011 and has been operating at scale since 2017. Of the other HIV services that were scaled-up in anticipation of DSD rollout, there was a high utilization of HIV status disclosure (83%) and participation in the treatment ‘buddy’ program (62.8%), while partner testing, community HIV community support group participation and targeted adherence counselling had lower rates of utilization (<50%). Fear of stigmatization remains entrenched in this setting as was previously reported by Kelly *et al*. and others [23–25] and was the most common reason (77%) for non-disclosure of HIV status. Thus, our findings suggest that while improvements have been made in many aspects of HIV service utilization, more resources are needed in other areas to bring the program up to readiness for full DSD implementation.

The goals of ART are to achieve and maintain durable viral suppression and restore immune function. From a prevention standpoint, sustained viral suppression is one of the most potent public health tools for interrupting the HIV transmission cycle (undetectable equals untransmittable) and limiting the emergence of drug resistance mutations [2–4,26]. To the best of our knowledge, this is the first study to assess virologic outcomes of HIV-infected patients receiving ART in Sierra Leone. Of the 429 patients who had VL measurements in the preceding 12 months, about two-thirds (64%) were virally suppressed, which was over twice the national rate of <30% achieved in 2018 [1].

Virologic failure remains a major barrier hindering the success of ART programs in SSA [1,6]. In our study, virologic failure was predicted by similar biological and behavioral factors that have been consistently observed in multiple studies from SSA [27–30], including immunosuppression (CD4 count <350 cells/mL), poor adherence, not received targeted adherence counselling, one-monthly versus multi-monthly ART dispensing, ATV/r-based ART and history of ART substitution.

Antiretroviral therapy substitution was most commonly carried out in the event of suspected HIV drug resistance (HIVDR). According to recent WHO reports, the prevalence of HIVDR is increasing globally, with countries in SSA being the worst affected [31]. The WHO has called for the integration of HIVDR monitoring and management strategies into broader HIV prevention and control efforts; however, Sierra Leone and similar resource-limited settings lack the laboratory capacity to do so [32]. Thus, we were unable to assess the prevalence and impact of HIVDR on treatment outcomes in this cohort. Nonetheless, we hypothesize that high rates of HIVDR could partly explain the high rates of virologic failure in this setting. Of note, in the only study to date from Sierra Leone characterizing HIVDR, we previously observed a high prevalence of acquired HIVDR (>95%) in an unrelated cohort of HIV-infected individuals (n = 151) aged >18 years at this same facility [12].

Over half (52%) of clinic attendees had been on ART for more than 12 months and were otherwise well but were classified as ‘unstable clients’. The commonest reasons were elevated VL (>1000 copies/mm^3^) (37.2%) and lack of VL measurement in the preceding 12 months (14.5%). This is a substantial proportion of patients of in HIV care and are the focus of current efforts aimed at improving clinical outcomes. Our analysis of services utilization and risk factors of poor virologic outcomes offers insights into strategies that could help this large pool of clients achieve better treatment outcomes and increase the number of stable patients. These include measures to increase clinic attendance, processes to streamline VL testing for clients, increasing adherence support for patients failing on ART and multi-monthly ART dispensing. More research is needed to identify factors and processes that could improve HIV services up and virologic outcomes in this setting.

Our study had strengths and weaknesses worthy of note. This was a quantitative analysis from a single (though largest) HIV treatment center in Sierra Leone, which may limit its generalizability. Secondly, analysis of potential barriers to accessing and utilization of HIV services and virologic outcomes were not exhaustive, due to the retrospective nature of the study design and limited data available for analysis. Another limitation was that we were unable to assess the implementation, service and client outcome measures of feasibility, acceptability, adoption (uptake), cost, efficiency of service delivery and client satisfaction – all of which are integral components of effective program planning, implementation and evaluation. Nonetheless, our findings add to the limited body of research into a relatively nascent but rapidly expanding approach to effective HIV care delivery in Sierra Leone and similar resource-limited settings in West Africa.

## Conclusion

In this pre-implementation analysis, we estimated that about one-third of recent adult HIV clinic attendees at the largest HIV treatment center in Sierra Leone qualified as stable for ART delivery based on the proposed DSD models. While there was high utilization of certain HIV services such as HIV status disclosure and treatment buddy program participation, there was low uptake in partner testing, HIV community support group participation and targeted adherence counselling. About two-thirds of patients with available viral load in the preceding 12 months were virally suppressed. Virologic failure was predicted by low CD4 (<350 cells/mm^3^), being on ritonavir-boosted atazanavir-based ART, one-monthly versus multi-monthly ART dispensing, history of ART switching, poor treatment adherence and not having received targeted adherence counselling. Our findings suggest that additional resources are needed to bring the program up to readiness for full DSD implementation.

## Paper context

There are limited data to help guide implementation of differentiated HIV service delivery (DSD) in Sierra Leone and other countries in sub-Saharan Africa. This pre-implementation analysis found that about one-third of HIV patients on ART were eligible for DSD. We identified gaps in HIV care (i.e. low partner testing, treatment ‘buddy’, program participation and significant proportion of patients with virologic failure) that need to be addressed in preparation for full implementation of DSD in Sierra Leone.
